# Impact of valvular surgery according to frailty risk in patients with infective endocarditis

**DOI:** 10.1002/clc.24268

**Published:** 2024-05-13

**Authors:** Carlos Diaz‐Arocutipa, Guillermo Moreno, Lourdes Vicent

**Affiliations:** ^1^ Unidad de Revisiones Sistemáticas y Meta‐análisis (URSIGET), Vicerrectorado de Investigación Universidad San Ignacio de Loyola Lima Peru; ^2^ Servicio de Cardiología, Hospital Universitario 12 de Octubre Madrid Spain; ^3^ Facultad de Enfermería, Fisioterapia y Podología Universidad Complutense de Madrid (UCM) Madrid Spain

**Keywords:** frailty, infective endocarditis, mortality, valvular surgery

## Abstract

**Background:**

Observational studies suggest that valvular surgery can reduce mortality in selected patients with infective endocarditis (IE). However, the benefit of this intervention according to frailty levels remains unclear. Our study aims to assess the effect of valvular surgery according to frailty status in this population.

**Methods:**

We performed a retrospective study using the 2016−2019 National Inpatient Sample database. Adult patients with a primary diagnosis of IE were included. Frailty was assessed using the Hospital Frailty Risk Score. Inverse probability of treatment weighting (IPTW) was used to balance baseline differences between groups.

**Results:**

A total of 53,275 patients with IE were included, with 18.3% underwent valvular surgery. The median age was 52 (34−68) years, with 41% females. Overall, 42.7% had low risk of frailty, 53.1% intermediate risk, and 4.2% high risk. After IPTW adjustment, in‐hospital mortality was similar both for the entire cohort between valvular and non‐valvular surgery groups (3.7% vs. 4.1%, *p* = .483), and low (1% vs. 0.9%, *p* = .952) or moderate (5.4% vs. 6%, *p* = .548) risk of frailty. However, patients at high risk of frailty had significantly lower in‐hospital mortality in the valvular surgery group (4.6% vs. 13.9%, *p* = .016). Renal replacement therapy was similar between groups across frailty status. In contrast, surgery was associated with increased use of mechanical circulatory support and pacemaker implantation.

**Conclusions:**

Our findings suggest that there was no difference in survival between valve surgery and medical management in patients at low/intermediate frailty risk, but not for high‐risk individuals.

## Graphical abstract

In adult patients with infective endocarditis, there was no difference in in‐hospital mortality between the valvular surgery and nonvalvular surgery group in patients with low and intermediate risk of frailty, except for high risk where mortality was lower in the operated group.

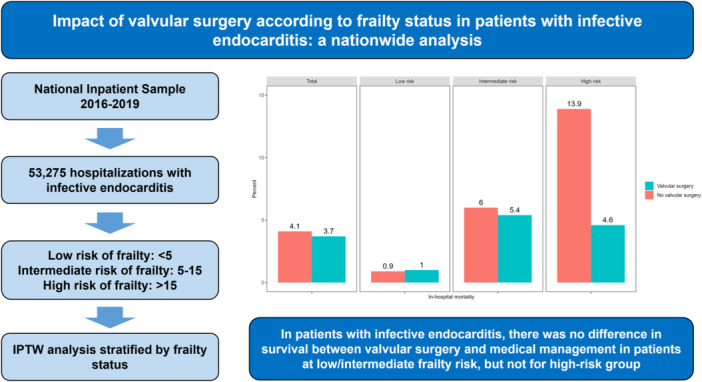

**Abbreviations**
AHRQ: Agency for Healthcare Research and Quality
CI: confidence interval
HFRS: Hospital Frailty Risk Score
ICD‐10: International Classification of Disease, Tenth Revision
IE: infective endocarditis
IPTW: inverse probability of treatment weighting
NIS: National Inpatient Sample
OR: odds ratio
SMD: standardized mean difference

## 1 INTRODUCTION

Infective endocarditis (IE) is a disease associated with high mortality.^1^ In recent decades, the epidemiology of IE has changed, with increasing patient age and complex cases associated with invasive procedures, including structural cardiac interventions^2^, ^3^ and healthcare‐associated cases.^4^

Frailty is a condition associated with increased vulnerability and low functional reserve, resulting in a very low adaptive response to external stressors.^5^ Frailty can occur at any time in life, although it is more common in older people.^6^ However, it has been described that the presence of frailty is associated with poor outcomes in several clinical scenarios.^7^, ^8^, ^9^ The prevalence of frailty varies depending on the context. In the general population, it can be more than 10%,^1^ but in a hospital setting, it can be much higher in patients with severe acute pathology and previous medical conditions.

To date, no studies have examined the role of frailty in patients with IE. In daily clinical practice, there is no systematic or comprehensive assessment of frailty as part of the decision‐making process for the management of these patients. The decision to treat IE surgically is based on subjective medical judgment and scales that do not specifically reflect the value of frailty.^10^, ^11^ Frailty may therefore have an impact on both the type of treatment patients with IE receive and their outcomes after treatment.

There are some patients with a surgical indication for IE who do not undergo surgery because of the presence of risk factors for complications or an unacceptably high perceived surgical risk.^12^ Frailty may be one of the factors influencing treatment decisions in the IE patient population. It is unclear whether a high degree of frailty should determine the conservative management of patients with IE, or whether this is a potentially salvageable group of patients with surgical treatment.

In this study, we analyzed data from a large cohort of patients diagnosed with IE to assess treatment received and prognosis based on their frailty status.

## 2 METHODS

### 2.1 Data source

Our retrospective cohort study used the National Inpatient Sample (NIS) database from 2016 to 2019, which is a publicly available database developed by the Agency for Healthcare Research and Quality (AHRQ) as part of the Healthcare Cost and Utilization Project. Each year the NIS database contains more than 7 million hospitalizations which represents a stratified sample of 20% of all‐payer hospital admissions from US hospitals. The NIS data used in this study are claims‐based and deidentified; thus, the study did not require Institutional Review Board approval and the requirement for written informed consent was waived.

### 2.2 Study population

Adult patients (≥18 years old) hospitalized with a primary diagnosis of IE were included, using the International Classification of Disease, Tenth Revision (ICD‐10) codes I330, I339, I38, and I39. Patients with missing data and those who died until the first day of admission were excluded. Neurological events or stroke were not considered as exclusion criteria.

### 2.3 Exposure

The main exposure variable was the occurrence of valvular surgery during hospitalization, which was identified by the following ICD‐10 procedure codes for left and/or right‐side surgery: 02QF*, 02QG*, 02QH*, 02QJ*, 02RF*, 02RG*, 02RH*, 02RJ*, and 02TH*.

### 2.4 Frailty

Frailty was defined according to the Hospital Frailty Risk Score.^13^ This tool has been previously validated to assess frailty using administrative health data based on ICD‐10 codes from 109 conditions. We expressed frailty as continuous data and categorized it as low risk (<5 points), intermediate risk (5–15 points), and high risk of frailty (>15 points).

### 2.5 Covariates

Information on patient characteristics such as age, sex, race, household income according to ZIP code, previous prosthetic valve, type of admission, weekend admission, length of hospital stay, expected primary payer, transfer out indicator, and total charges were recorded. Likewise, the comorbidity burden was assessed using the modified version of the Elixhauser Comorbidity Index. Hospital characteristics, including hospital bed size, location of hospital, region of hospital, and ownership of hospital were also collected.

### 2.6 Outcomes

The primary outcome was in‐hospital mortality and secondary outcomes were septic shock, need for renal replacement therapy, mechanical circulatory support (intra‐aortic balloon pump, percutaneous ventricular assist device, or extracorporeal membrane oxygenation), and transvenous pacemaker implantation. All diagnostic and procedural ICD‐10 codes for outcomes are available in Supporting Information: Table 1.

### 2.7 Statistical analysis

Categorical data were expressed using absolute and relative frequencies, while continuous data as median (percentile 25−percentile 75). The Chi‐square test with the Rao‐Scott correction was used to assess the association between categorical data and the Wilcoxon rank‐sum test to compare continuous and categorical data. Inverse probability of treatment weighting (IPTW) was used to address confounding bias on the effect of valvular surgery on all outcomes. We compared the balance with and without weighting of covariates between valvular surgery and nonvalvular surgery groups using the standardized mean difference (SMD), considering an appropriate balance when the SMD < 0.1. Using a binary logistic regression model, the effect of valvular surgery was assessed by estimating the odds ratio (OR) with its 95% confidence interval (CI). All analyses were stratified according to frailty levels (low risk, intermediate risk, and high risk), using sampling weights to represent national estimates as recommended by AHRQ. In addition, we conducted a restricted cubic spline regression model (using three knots) to assess the association between valvular surgery and in‐hospital mortality according to frailty score (as continuous data), adjusting for demographics, comorbidity burden, and hospital characteristics. We used the R 4.3.2 software (R Foundation for Statistical Computing, Vienna, Austria) for all analyses. A two‐tailed *p* < .05 was considered statistically significant.

## 3 RESULTS

### 3.1 Patient and hospital characteristics

**Figure 1:**
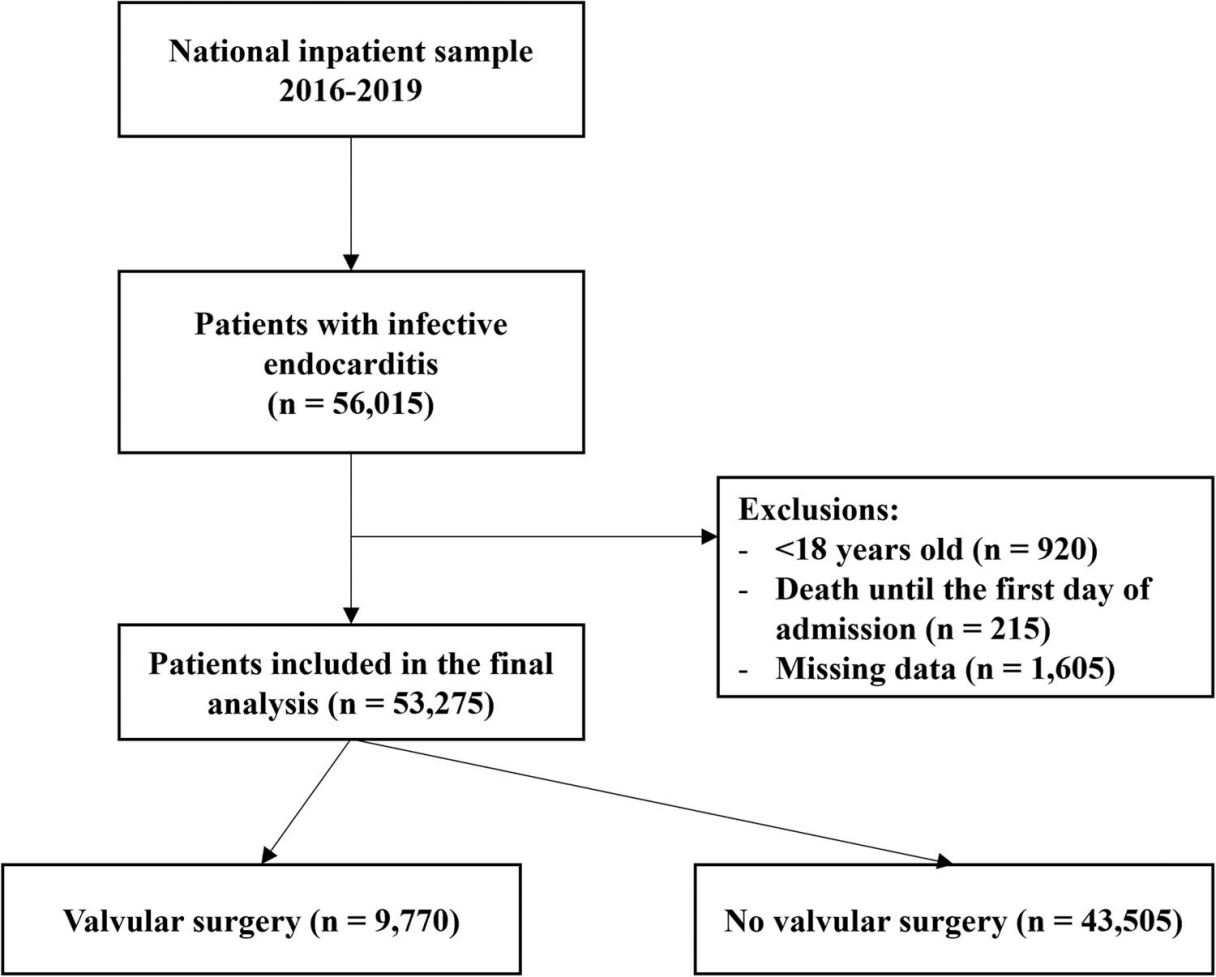
Flow diagram for the selection of study participants.

Of 56,015 patients with IE, 53 275 were included in the final analysis (Figure 1). Characteristics of the total population and for each level of frailty risk are shown in Table 1. The median age was 52 years (IQR: 34−68), 59% were male, and 74% were white. Operated patients were younger overall and for intermediate and high risk of frailty. The median length of hospital stay was 8 days (4–16). Patients in the valvular surgery group had longer hospitalization times in the whole population (median 17 vs. 7 days, *p* < .001) and for each level of frailty risk. The most common comorbidities were valvular disease (55%), hypertension (50%), and fluid and electrolyte disorders (41%) (Supporting Information: Table 2). The median score of the Elixhauser Comorbidity Index was 5 (3 − 6), with significantly higher scores in total operated patients (median 6 vs. 4, *p* < .001) and in those at low and intermediate risk of frailty. Only 9% of patients had a previous prosthetic valve, with a lower proportion in all operated patients compared to non‐operated patients (2% vs. 11%, *p* < .001), and for those at low (3% vs. 12%, *p* < .001) and intermediate (2% vs. 10%, *p* < .001) risk of frailty. Overall, 39% of patients were identified as intravenous drug users. The majority of patients were hospitalized in a large‐bed hospital (56%), urban teaching hospital (76%), and private non‐profit hospital (77%). The type of admission was nonelective in 90% of cases and 61% were not transferred to another hospital. Valvular surgery was performed in 18.3% of cases and, in the majority (84%), the operated valve was only on the left side. Left heart valves were the most commonly operated on. In 15% of cases, one valve was operated on, and in 3% two valves were intervened. Specifically, we found that 55% of patients underwent surgery on the aortic valve, making it the most commonly treated heart valve. This was closely followed by the mitral valve (46%). Surgery on the pulmonary valve was the least common, with only 1% of patients having surgery on this valve. Tricuspid valve surgery was performed in 16% of cases. Overall, 42.7% had low risk of frailty, 53.1% intermediate risk of frailty, and 4.2% high risk of frailty. Baseline covariates were well balanced after IPTW adjustment (Supporting Information: Figure 1).

**Table 1:** Characteristics of included patients according to valvular surgery status and stratified by risk of frailty.

Characteristic	Total	Valvular surgery		*p* Value	Low risk		*p* Value	Intermediate risk		*p* Value	High risk		*p* Value
					Valvular surgery			Valvular surgery			Valvular surgery		
		No	Yes		No	Yes		No	Yes		No	Yes	
Weighted hospitalizations	53 275	43 505	9770		19 895	2875		21 990	6305		1620	590	
Age (years)^a^	52 (34−68)	52 (34−69)	52 (37−63)	.002	43 (31−62)	48 (35−61)	<.001	58 (38−72)	52 (37−63)	<.001	68 (56−81)	57 (43−67)	<.001
Female sex	21 945 (41%)	18 535 (43%)	3410 (35%)	<.001	8340 (42%)	980 (34%)	<.001	9525 (43%)	2215 (35%)	<.001	670 (41%)	215 (36%)	.351
Race				<.001			.283			.019			.258
White	39 680 (74%)	32 760 (75%)	6920 (71%)		15 285 (77%)	2105 (73%)		16 265 (74%)	4425 (70%)		1210 (75%)	390 (66%)	
Black	5340 (10%)	4230 (10%)	1110 (11%)		1555 (8%)	250 (9%)		2465 (11%)	750 (12%)		210 (13%)	110 (19%)	
Hispanic	4020 (8%)	3225 (7%)	795 (8%)		1575 (8%)	275 (10%)		1550 (7%)	485 (8%)		100 (6%)	35 (6%)	
Other	4235 (8%)	3290 (8%)	945 (10%)		1480 (7%)	245 (9%)		1710 (8%)	645 (10%)		100 (6%)	55 (9%)	
Household income				.010			.032			.241			.210
Quartile 1	17 500 (33%)	14 465 (33%)	3035 (31%)		6480 (33%)	830 (29%)		7545 (34%)	2040 (32%)		440 (27%)	165 (28%)	
Quartile 2	13 740 (26%)	11 365 (26%)	2375 (24%)		5310 (27%)	700 (24%)		5640 (26%)	1560 (25%)		415 (26%)	115 (19%)	
Quartile 3	12 230 (23%)	9870 (23%)	2360 (24%)		4555 (23%)	710 (25%)		4925 (22%)	1455 (23%)		390 (24%)	195 (33%)	
Quartile 4	9805 (18%)	7805 (18%)	2000 (20%)		3550 (18%)	635 (22%)		3880 (18%)	1250 (20%)		375 (23%)	115 (19%)	
Elective admission	5170 (10%)	4055 (9%)	1115 (11%)	.005	2275 (11%)	475 (17%)	<.001	1680 (8%)	625 (10%)	.009	100 (6%)	15 (3%)	.129
Admission on weekend	11 885 (22%)	9915 (23%)	1970 (20%)	.012	4310 (22%)	500 (17%)	.019	5190 (24%)	1305 (21%)	.031	415 (26%)	165 (28%)	.620
Length of hospital stay (days)^a^	8 (4−16)	7 (4−13)	17 (12−27)	<.001	5 (3−9)	13 (9−20)	<.001	8 (5−15)	18 (13−29)	<.001	12 (8−18)	24 (18−36)	<.001
Expected primary payer				<.001			<.001			<.001			<.001
Medicare	19 300 (36%)	16 455 (38%)	2845 (29%)		5225 (26%)	640 (22%)		10 175 (46%)	1965 (31%)		1055 (65%)	240 (41%)	
Medicaid	17 155 (32%)	14 410 (33%)	2745 (28%)		7800 (39%)	790 (27%)		6320 (29%)	1810 (29%)		290 (18%)	145 (25%)	
Private	10 725 (20%)	7610 (17%)	3115 (32%)		4135 (21%)	1095 (38%)		3260 (15%)	1870 (30%)		215 (13%)	150 (25%)	
Other	6095 (11%)	5030 (12%)	1065 (11%)		2735 (14%)	350 (12%)		2,235 (10%)	660 (10%)		60 (4%)	55 (9%)	
Total charges^a^	67 222 (31 567−177 675)	51 800 (26 787−101 171)	306 341 (214 967−476 964)	<.001	36 059 (20 017−67 880)	251 337 (174 388−364 899)	<.001	65 652 (36 446−126 371)	330 043 (228 791−530 370)	<.001	107 102 (61 292−194 382)	404 936 (295 112−577 079)	<.001
Bed size of hospital				<.001			<.001			<.001			<.001
Small	9305 (17%)	8710 (20%)	595 (6%)		4415 (22%)	240 (8%)		4060 (18%)	330 (5%)		235 (15%)	25 (4%)	
Medium	14 155 (27%)	12 145 (28%)	2010 (21%)		5475 (28%)	640 (22%)		6150 (28%)	1260 (20%)		520 (32%)	110 (19%)	
Large	29 815 (56%)	22 650 (52%)	7165 (73%)		10 005 (50%)	1995 (69%)		11 780 (54%)	4,715 (75%)		865 (53%)	455 (77%)	
Location of hospital				<.001			<.001			<.001			.065
Rural	3575 (7%)	3440 (8%)	135 (1%)		1850 (9%)	45 (2%)		1530 (7%)	85 (1%)		60 (4%)	5 (1%)	
Urban nonteaching	9415 (18%)	8560 (20%)	855 (9%)		4160 (21%)	335 (12%)		4185 (19%)	475 (8%)		215 (13%)	45 (8%)	
Urban teaching	40 285 (76%)	31 505 (72%)	8780 (90%)		13 885 (70%)	2495 (87%)		16 275 (74%)	5745 (91%)		1345 (83%)	540 (92%)	
Region of hospital				.018			.198			.006			.603
Northeast	12 210 (23%)	10 135 (23%)	2075 (21%)		4960 (25%)	820 (29%)		4870 (22%)	1175 (19%)		305 (19%)	80 (14%)	
Midwest	11 270 (21%)	9025 (21%)	2245 (23%)		3745 (19%)	475 (17%)		4820 (22%)	1580 (25%)		460 (28%)	190 (32%)	
South	20 430 (38%)	16 820 (39%)	3610 (37%)		7805 (39%)	1070 (37%)		8420 (38%)	2320 (37%)		595 (37%)	220 (37%)	
West	9365 (18%)	7525 (17%)	1840 (19%)		3385 (17%)	510 (18%)		3880 (18%)	1230 (20%)		260 (16%)	100 (17%)	
Ownership of hospital				.002			.012			.047			.873
Government, nonfederal	7075 (13%)	5950 (14%)	1125 (12%)		2775 (14%)	280 (10%)		2975 (14%)	770 (12%)		200 (12%)	75 (13%)	
Private, non‐profit	40 895 (77%)	33 095 (76%)	7800 (80%)		14 930 (75%)	2305 (80%)		16 885 (77%)	5040 (80%)		1280 (79%)	455 (77%)	
Private, investor‐own	5305 (10%)	4460 (10%)	845 (9%)		2190 (11%)	290 (10%)		2130 (10%)	495 (8%)		140 (9%)	60 (10%)	
Transfer in indicator				<.001			<.001			<.001			<.001
Not a transfer	37 280 (70%)	32 715 (75%)	4565 (47%)		15 655 (79%)	1455 (51%)		15 955 (73%)	2845 (45%)		1105 (69%)	265 (45%)	
Different acute care hospital	13 535 (25%)	8810 (20%)	4,725 (48%)		3485 (18%)	1225 (43%)		4950 (23%)	3,210 (51%)		375 (23%)	290 (49%)	
Another type of health facility	2295 (4%)	1840 (4%)	455 (5%)		705 (4%)	185 (6%)		1005 (5%)	235 (4%)		130 (8%)	35 (6%)	
Transfer out indicator				<.001			<.001			<.001			.002
Not a transfer	32 670 (61%)	27 115 (62%)	5555 (57%)		14 160 (71%)	2,030 (71%)		12 390 (56%)	3,400 (54%)		565 (35%)	125 (21%)	
Different acute care hospital	6295 (12%)	5815 (13%)	480 (5%)		2705 (14%)	145 (5%)		2930 (13%)	300 (5%)		180 (11%)	35 (6%)	
Another type of health facility	14 310 (27%)	10 575 (24%)	3735 (38%)		3030 (15%)	700 (24%)		6670 (30%)	2,605 (41%)		875 (54%)	430 (73%)	
Prosthetic valve	4910 (9%)	4700 (11%)	210 (2%)	<.001	2395 (12%)	85 (3%)	<.001	2205 (10%)	115 (2%)	<.001	100 (6%)	10 (2%)	.056
Elixhauser Comorbidity Index^a^	5.00 (3.00−6.00)	4.00 (3.00−6.00)	6.00 (4.00−7.00)	<.001	3.00 (2.00−5.00)	4.00 (3.00−6.00)	<.001	5.00 (4.00−7.00)	6.00 (5.00−8.00)	<.001	6.00 (5.00−8.00)	7.00 (5.50−8.00)	.087
Aortic valve surgery	5330 (10%)	0 (0%)	5330 (55%)	<.001	0 (0%)	1560 (54%)	<.001	0 (0%)	3465 (55%)	<.001	0 (0%)	305 (52%)	<.001
Mitral valve surgery	4500 (8%)	0 (0%)	4500 (46%)	<.001	0 (0%)	1215 (42%)	<.001	0 (0%)	2960 (47%)	<.001	0 (0%)	325 (55%)	<.001
Pulmonary valve surgery	70 (0%)	0 (0%)	70 (1%)	<.001	0 (0%)	25 (1%)	<.001	0 (0%)	45 (1%)	<.001	0 (0%)	0 (0%)	
Tricuspid valve surgery	1540 (3%)	0 (0%)	1540 (16%)	<.001	0 (0%)	455 (16%)	<.001	0 (0%)	1030 (16%)	<.001	0 (0%)	55 (9%)	<.001
Injection drug user	20 615 (39%)	17 595 (40%)	3020 (31%)	<.001	9815 (49%)	885 (31%)	<.001	7490 (34%)	1975 (31%)	.069	290 (18%)	160 (27%)	.034
Number of operated valves				<.001			<.001			<.001			<.001
0	43 505 (81.7%)	43 505 (100%)	0 (0%)		19 895 (100%)	0 (0%)		21 990 (100%)	0 (0%)		1620 (100%)	0 (0%)	
1	8175 (15.3%)	0 (0%)	8175 (84%)		0 (0%)	2500 (87%)		0 (0%)	5175 (82%)		0 (0%)	500 (85%)	
2	1520 (2.9%)	0 (0%)	1520 (16%)		0 (0%)	370 (13%)		0 (0%)	1065 (17%)		0 (0%)	85 (14%)	
3	75 (0.1%)	0 (0%)	75 (1%)		0 (0%)	5 (0.2%)		0 (0%)	65 (1%)		0 (0%)	5 (0.8%)	
Valve procedure location													
Left‐sided surgery	8180 (84%)	0 (0%)	8180 (84%)		‐	2395 (83%)		‐	5250 (83%)		‐	535 (91%)	
Right‐sided surgery	1210 (12%)	0 (0%)	1210 (12%)		‐	405 (14%)		‐	755 (12%)		‐	50 (8%)	
Left and right side surgery	380 (4%)	0 (0%)	380 (4%)		‐	75 (3%)		‐	300 (5%)		‐	5 (1%)	

^a^ Median (interquartile range).

### 3.2 Primary outcome

**Figure 2:**
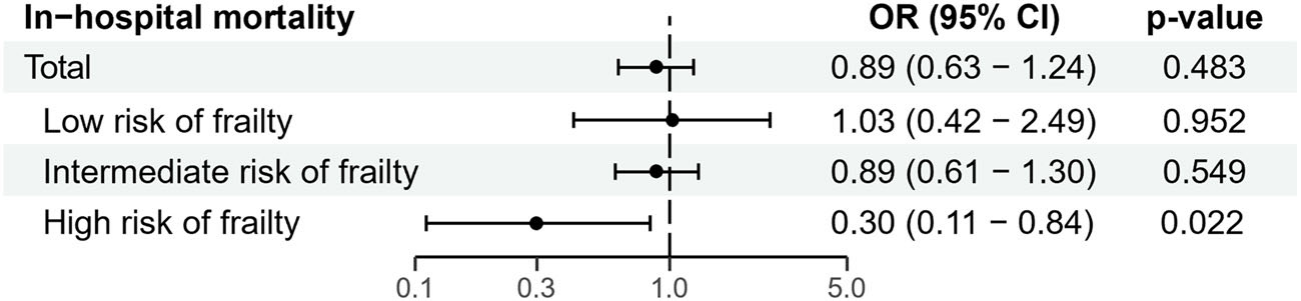
Forest plot showing the adjusted ORs with their 95% CIs for the effect of valvular surgery on in‐hospital mortality according to frailty status. CI, confidence interval; OR, odds ratio.

**Figure 3:**
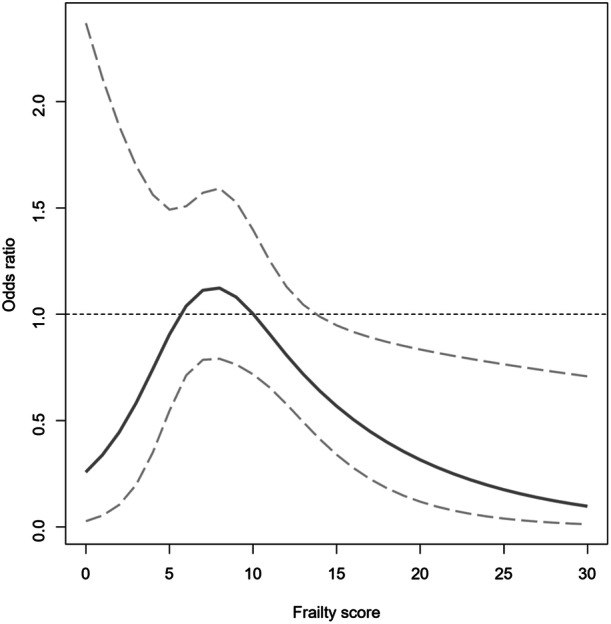
Effect of valvular surgery on in‐hospital mortality according to frailty score using a restricted cubic spline regression model adjusted for demographics, comorbidity burden, and hospital characteristics. Blue line indicates odds ratio and gray lines indicate 95% confidence interval.

After IPTW adjustment, there was no difference in in‐hospital mortality between patients with and without valve surgery (3.7% vs. 4.1%, OR 0.89, 95% CI 0.63−1.24, *p* = .483) (Table 2 and Figure 2). Similarly, the risk of in‐hospital mortality was comparable in patients with low (1% vs. 0.9%, OR 1.03, 95% CI 0.42−2.49, *p* = .952) and intermediate (5.4% vs. 6%, OR 0.89, 95% CI 0.61−1.30, *p* = .549) risk of frailty, irrespective of surgery. In contrast, in patients at high risk of frailty, in‐hospital mortality was significantly lower in operated cases compared to non‐operated cases (4.6% vs. 13.9%, OR 0.30, 95% CI 0.11−0.84, *p* = .022) (Table 2 and Figure 2). In addition, a nonlinear relationship was found between the frailty score and the effect of valve surgery on in‐hospital mortality, observing that when the frailty score was greater than 15, valvular surgery had a significant beneficial effect (Figure 3).

**Table 2:** Weighted outcomes according to valvular surgery status and stratified by risk of frailty.

Characteristic	Total		*p* Value	Low risk		*p* Value	Intermediate risk		*p* Value	High risk		*p* Value
	Valvular surgery			Valvular surgery			Valvular surgery			Valvular surgery		
	No	Yes		No	Yes		No	Yes		No	Yes	
In‐hospital mortality	4.1%	3.7%	.483	0.9%	1.0%	.952	6.0%	5.4%	.548	13.9%	4.6%	.016
Septic shock	2.6%	6.4%	<.001	0.3%	2.1%	<.001	4.1%	9.1%	<.001	7.9%	9.0%	.727
Renal replacement therapy	4.7%	4.2%	.457	1.2%	0.5%	.039	7.3%	5.9%	.102	7.1%	13.0%	.190
Mechanical circulatory support	0.4%	3.6%	<.001	0.1%	2.2%	<.001	0.7%	4.5%	<.001	0.0%	3.5%	<.001
Pacemaker implantation	0.4%	10.6%	<.001	0.3%	11.4%	<.001	0.5%	10.1%	<.001	0.4%	9.9%	<.001

### 3.3 Secondary outcomes

In the adjusted analysis, patients who underwent valvular surgery had a higher proportion of septic shock compared to those who did not (6.4% vs. 2.6%, *p* < .001) (Table 2). Similarly, septic shock was more frequent in operated patients with low (2.1% vs. 0.3%, *p* < .001) and intermediate (9.1% vs. 4.1%, *p* < .001) risk of frailty. However, this complication was similar in patients with high frailty risk, irrespective of surgery status (9% vs. 7.9%, *p* = .727). Overall, renal replacement therapy was not different between operated and non‐operated patients (4.2% vs. 4.7%, *p* = .457). This was also found in patients with intermediate (5.9% vs. 7.3%, *p* = .102) and high (13% vs. 7.1%, *p* = .190) risk of frailty. In addition, operated patients presented significantly higher use of mechanical circulatory support (3.6% vs. 0.4%, *p* < .001) and pacemaker implantation (10.6% vs. 0.4%, *p* < .001) considering the whole cohort and for each frailty risk level (Table 2).

## 4 DISCUSSION

In this large study, using a nationally representative sample of patients with IE, we have for the first time analyzed the role of frailty in the prognosis of patients with IE in accordance with valve surgery treatment. Frailty is very common in patients with IE, and more than half of the patients were at intermediate or high risk of frailty. Mortality was high in IE patients, especially in those with a high risk of frailty. However, compared with other frailty statuses, the group of patients with high frailty risk had a superior survival benefit from IE surgical treatment. Frailty should be assessed, but it should not be the sole factor in denying intervention for patients with IE who require valve surgery.

### 4.1 Comorbidities, frailty, and treatment decisions

The increasing prevalence of comorbidities in patients with IE makes it necessary to consider the role of treatment indications and the expected benefit of invasive interventions such as cardiac surgery, which is still controversial in many cases.^3^, ^12^, ^14^, ^15^ A high degree of comorbidity has led to a lower indication for surgery, especially in older patients.^3^ As expected, comorbidities were higher in patients with intermediate and high frailty risk, and those with high frailty risk were older. In addition, the level of comorbidity was higher in patients undergoing IE valve surgery, as reflected by the Elixhauser Comorbidity Index. Previous valvular heart disease played a significant role, with almost three out of four patients undergoing surgery for IE having a history of valvular heart disease. In recent decades, degenerative valve disease has become a very common predisposing factor for IE.^16^ We have found that in our cohort, prior organic valve involvement appears to play an important role in the decision to operate for IE. In this context, it is likely that previous comorbidities may have played a role in the severity and presentation of IE. This may have led to a greater indication for valve surgery in IE patients.

The proportion of women was similar in the three frailty states (around 40%), but was significantly lower in the group of patients undergoing valve surgery for IE, which has also been documented in previous studies.^15^, ^17^, ^18^, ^19^

We found a lower frequency of patients undergoing cardiac surgery compared to other series.^3^, ^15^ However, it is important to note that this is a national administrative database with a very large number of cases (>50 000) and that previous registries probably did not include cases of lesser severity that were managed differently, outpatient or in less complex centers.

### 4.2 Clinical outcomes according to valvular surgery and frailty status

We found that in‐hospital mortality in patients with IE was around 4%. In other studies, the mortality was variable, for example in the EURO‐ENDO registry the overall mortality was 17%.^20^ In our study, about 40% of patients had a transfer to another center. These may have been low‐risk cases, or without an indication for valve surgery, or where valve surgery was not performed because of an unacceptably high surgical risk. This high number of transferred patients may have influenced the observed mortality, which was lower than in other series.^21^, ^22^ In a previous study, Hémar V et al found that poor functional status in elderly patients predicted mortality in those undergoing surgery. In this sense, it would be important to individualize each case, because in fact the highest mortality in this cohort of patients occurred in the group of patients who did not undergo valvular surgery.^15^ In addition, the functional status of patients may be improved through targeted interventions such as rehabilitation, nutrition and other comprehensive geriatric care.

In terms of the feared risk of complications associated with surgery and postoperative ICU care, we observed a tendency for these to occur more often in patients with a high risk of frailty, even in cases where conservative management was performed. However, patients who underwent valve surgery for IE had a higher need for permanent cardiac pacing, but this was comparable in all three frailty states. This complication is likely to be related to the type of indication for surgery (i.e., periannular extension) or the surgical technique itself, and the incidence of this complication has been similar in previous studies.^23^

Three out of four patients with a high risk of frailty who underwent surgery were transferred to another healthcare facility instead of being discharged home. This is a significant finding, highlighting the importance of assessing high frailty cases and implementing a postcardiac surgery care plan that includes early rehabilitation. The implementation of specialized measures, such as physiotherapy, nutrition support, and cognitive stimulation, may increase the likelihood of patients being discharged home, reducing healthcare costs and improving their quality of life. Studies specifically examining the impact of frailty on postoperative recovery would be of interest.

### 4.3 Clinical implications

Frailty means a reduced compensatory capacity to adapt to acute stress situations,^24^ such as a serious illness like IE. Frailty has been associated with increased overall mortality and hospital complications.^25^, ^26^ Valvular surgery for IE is technically complex and associated with high mortality.^27^ Despite this risk, we found that patients with IE and a high risk of frailty benefited most from valve surgery for IE, with a risk of postoperative complications comparable to other frailty statuses (low or moderate). It is possible that the lower biological reserve of these patients, coupled with a potentially more severe clinical presentation of IE due to previous comorbidities, may make this patient population an eligible group for consideration of valve surgery for IE. However, it is important that each case is carefully assessed in a multidisciplinary team decision,^12^ taking into account the wishes of the patient and family. Although frailty should not be a limiting factor in indicating surgery in these patients, its assessment may be useful in identifying a group of patients at high risk of mortality who may benefit from closer monitoring. There are other important questions, such as the timing of surgery in these patients: in the acute phase or delayed intervention. Future research should investigate this point.

It is important to consider that patients with high levels of frailty may be deemed too high risk for surgery and referred to palliative care instead. Conversely, patients with lower levels of frailty and higher chances of survival may have been prioritized for surgical intervention. Our findings may have been impacted by self‐selection bias. As mentioned, it is important to consider these limitations when interpreting the results. It is not recommended to assume the benefits of surgery in patients with high frailty without further investigation. Furthermore, this study offers valuable insights into the management of endocarditis in frail patients and emphasizes the necessity for individualized treatment approaches based on careful risk assessment.

Frailty can significantly contribute to overall risk, yet it is currently not considered in widely used risk calculators such as EUROSCORE or STS. While current risk assessment tools primarily focus on traditional risk factors, our study emphasizes the necessity of a more comprehensive approach that includes frailty assessment in preoperative protocols. Our findings highlight the impact of frailty on patient outcomes and support the integration of frailty assessment into clinical practice to improve risk prediction and optimize patient care strategies.

### 4.4 Limitations

There are a number of limitations that need to be acknowledged. First, our study was based on retrospective data extracted from the NIS database, which has inherent limitations related to the accuracy and completeness of the data and the potential for bias in coding practices across institutions and over time. Thus, the reliability of the observed associations could be affected by misclassification. Second, as a clinical‐administrative database, other prognostic variables, such as medical therapies or echocardiography variables, were missing. There is a lack of information on the indication for valvular surgery, which could help to understand the clinical scenarios where surgery could be performed in patients with high frailty. In addition, there is no information on causative micro‐organisms. Finally, because the database only contains information collected while hospitalized, it was not possible to assess medium‐ and long‐term outcomes. It is also important to note that the number of patients in the high frailty risk category undergoing surgery was slightly lower than in other categories, which may have limited the statistical power of some comparisons.

## 5 CONCLUSION

In patients at high risk of frailty who are admitted with a primary diagnosis of IE, the decision to perform valve surgery should be made carefully, as a benefit in reducing mortality has been observed in this category of patients. Patients with EI who underwent valve surgery had a greater need for pacemaker implantation and mechanical circulatory support, but these complications occurred similarly in all three frailty states. However, prospective studies evaluating objective clinical measures of frailty and interventions that mitigate the effects on clinical outcomes are needed.

## AUTHOR CONTRIBUTIONS

Carlos Diaz‐Arocutipa involved in concept/design. Carlos Diaz‐Arocutipa involved in data acquisition. Carlos Diaz‐Arocutipa, Guillermo Moreno, and Lourdes Vicent involved in data analysis/interpretation. Carlos Diaz‐Arocutipa and Lourdes Vicent drafted the article. Carlos Diaz‐Arocutipa, Guillermo Moreno, and Lourdes Vicent critically revised the article. Carlos Diaz‐Arocutipa, Guillermo Moreno, and Lourdes Vicent approved the article.

## CONFLICT OF INTEREST STATEMENT

The author declare no conflict of interest.

## Supporting information

Supporting information.

## Data Availability

Data are available in a public, open access repository. National Inpatient Sample is available online at https://hcup-us.ahrq.gov/databases.jsp.

## References

[1] Chen H , Zhan Y , Zhang K , et al. The global, regional, and national burden and trends of infective endocarditis from 1990 to 2019: results from the global burden of disease study 2019. Front Med. 2022;9:774224. 10.3389/fmed.2022.774224 PMC8959916 35355601

[2] Durante‐Mangoni E . Current features of infective endocarditis in elderly patients: results of the international collaboration on endocarditis prospective cohort study. Arch Intern Med. 2008;168(19):2095‐2103. 18955638 10.1001/archinte.168.19.2095

[3] Nagai T , Takase Y , Hamabe A , Tabata H . Observational study of infective endocarditis at a community‐based hospital: dominance of elderly patients with comorbidity. Intern Med. 2018;57(3):301‐310. 29225254 10.2169/internalmedicine.9274-17 PMC5827306

[4] Musci T , Grubitzsch H . Healthcare‐associated infective endocarditis‐surgical perspectives. J Clin Med. 2022;11(17):4957. 36078887 10.3390/jcm11174957 PMC9457102

[5] Xue QL . The frailty syndrome: definition and natural history. Clin Geriatr Med. 2011;27(1):1‐15. 21093718 10.1016/j.cger.2010.08.009 PMC3028599

[6] O'Caoimh R , Sezgin D , O'Donovan MR , et al. Prevalence of frailty in 62 countries across the world: a systematic review and meta‐analysis of population‐level studies. Age Ageing. 2021;50(1):96‐104. 33068107 10.1093/ageing/afaa219

[7] Esteve‐Pastor MA , Martín E , Alegre O , et al. Impact of frailty and atrial fibrillation in elderly patients with acute coronary syndromes. Eur J Clin Invest. 2021;51(5):e13505. 33529346 10.1111/eci.13505

[8] Nowak W , Kowalik I , Nowicki M , Cichocki T , Stępińska J . The impact of frailty on in‐hospital complications in elderly patients with acute coronary syndrome. J Geria Cardiol. 2023;20(3):174‐184. 10.26599/1671-5411.2023.03.003 PMC10114198 37091258

[9] Arnold SV , Zhao Y , Leon MB , et al. Impact of frailty and prefrailty on outcomes of transcatheter or surgical aortic valve replacement. Circ Cardiovas Intervent. 2022;15(1):e011375. 10.1161/CIRCINTERVENTIONS.121.011375 35041454

[10] Thourani VH , Badhwar V , Shahian DM , et al. The society of thoracic surgeons adult cardiac surgery database: 2017 update on research. Ann Thorac Surg. 2017;104(1):22‐28. 28577849 10.1016/j.athoracsur.2017.05.013

[11] Ad N , Holmes SD , Patel J , Pritchard G , Shuman DJ , Halpin L . Comparison of euroscore ii, original euroscore, and the society of thoracic surgeons risk score in cardiac surgery patients. Ann Thorac Surg. 2016;102(2):573‐579. 27112651 10.1016/j.athoracsur.2016.01.105

[12] Delgado V , Ajmone Marsan N , de Waha S , et al. 2023 ESC Guidelines for the management of endocarditis. Eur Heart J. 2023;44(39):3948‐4042. 37622656 10.1093/eurheartj/ehad193

[13] Gilbert T , Neuburger J , Kraindler J , et al. Development and validation of a hospital frailty risk score focusing on older people in acute care settings using electronic hospital records: an observational study. The Lancet. 2018;391(10132):1775‐1782. 10.1016/S0140-6736(18)30668-8 PMC5946808 29706364

[14] Delahaye F , de Gevigney G . Is surgery useful in infective endocarditis? Arch Cardiovasc Dis. 2008;101(11‐12):685‐686. 19059562 10.1016/j.acvd.2008.10.005

[15] Hémar V , Camou F , Roubaud‐Baudron C , et al. The mortality of infective endocarditis with and without surgery in elderly (moise) study. Clin Infect Dis. 2023;77(10):1440‐1448. 37369092 10.1093/cid/ciad384

[16] Fernández‐Hidalgo N , Tornos Mas P . Epidemiología de la endocarditis infecciosa en España en los últimos 20 años. Rev Esp Cardiol. 2013;66(9):728‐733. 24773679 10.1016/j.rec.2013.05.002

[17] Armiñanzas C , Fariñas‐Alvarez C , Zarauza J , et al. Role of age and comorbidities in mortality of patients with infective endocarditis. Eur J Intern Med. 2019;64:63‐71. 30904433 10.1016/j.ejim.2019.03.006

[18] Bannay A , Hoen B , Duval X , et al. The impact of valve surgery on short‐ and long‐term mortality in left‐sided infective endocarditis: do differences in methodological approaches explain previous conflicting results? Eur Heart J. 2011;32(16):2003‐2015. 19208650 10.1093/eurheartj/ehp008

[19] Sambola A , Fernández‐Hidalgo N , Almirante B , et al. Sex differences in native‐valve infective endocarditis in a single tertiary‐care hospital. Am J Cardiol. 2010;106(1):92‐98. 20609654 10.1016/j.amjcard.2010.02.019

[20] Habib G , Erba PA , Iung B , et al. Clinical presentation, aetiology and outcome of infective endocarditis. Eur Heart J. 2019;40(39):3222‐3232. 31504413 10.1093/eurheartj/ehz620

[21] Ahtela E , Oksi J , Porela P , Ekström T , Rautava P , Kytö V . Trends in occurrence and 30‐day mortality of infective endocarditis in adults: population‐based registry study in Finland. BMJ Open. 2019;9(4):e026811. 10.1136/bmjopen-2018-026811 PMC6500343 31005935

[22] Wallace SM . Mortality from infective endocarditis: clinical predictors of outcome. Heart. 2002;88(1):53‐60. 12067945 10.1136/heart.88.1.53 PMC1767155

[23] Hill TE , Kiehl EL , Shrestha NK , et al. Predictors of permanent pacemaker requirement after cardiac surgery for infective endocarditis. Eur Heart J Acute Cardiovasc Care. 2021;10(3):329‐334. 33974691 10.1177/2048872619848661

[24] Kojima G , Iliffe S , Walters K . Frailty index as a predictor of mortality: a systematic review and meta‐analysis. Age Ageing. 2018;47(2):193‐200. 29040347 10.1093/ageing/afx162

[25] Peng Y , Zhong GC , Zhou X , Guan L , Zhou L . Frailty and risks of all‐cause and cause‐specific death in community‐dwelling adults: a systematic review and meta‐analysis. BMC Geriatr. 2022;22(1):725. 36056319 10.1186/s12877-022-03404-w PMC9437382

[26] Alegre O , Formiga F , López‐Palop R , et al. An easy assessment of frailty at baseline independently predicts prognosis in very elderly patients with acute coronary syndromes. J Am Med Dir Assoc. 2018;19(4):296‐303. 29153753 10.1016/j.jamda.2017.10.007

[27] Prendergast BD , Tornos P . Surgery for infective endocarditis: who and when? Circulation. 2010;121(9):1141‐1152. 20212293 10.1161/CIRCULATIONAHA.108.773598

