# Supplementary material for: Impact of valvular surgery according to frailty risk in patients with infective endocarditis
Source: Clin Cardiol. 2024 May 13;47(5):e24268. doi: 10.1002/clc.24268 (PMC11091451; doi:10.1002/clc.24268)
Supplement: Supplementary file 1 — Supporting information. [file CLC-47-e24268-s001.docx]

**SUPPLEMENTARY DATA**

**Supplemental Table 1. Search ICD-10 codes**

| **Diagnoses/Procedures** | **Codes** |
| --- | --- |
| Infective endocarditis | I330, I339, I38, I39 |
| Valvular surgery | 02QF*, 02QG*, 02QH*, 02QJ*, 02RF*, 02RG*, 02RH*, 02RJ*, 02TH* |
| Prosthetic valve | Z952, Z953 |
| Septic shock | R6521 |
| Pacemaker implantation | 0JH80PZ, 0JH63PZ, 0JH60PZ, 0JH836Z, 0JH806Z, 0JH636Z, 0JH606Z, 0JH835Z, 0JH805Z, 0JH635Z, 0JH605Z, 0JH834Z, 0JH804Z, 0JH634Z, 0JH604Z, 0JH83PZ, 02H64JZ, 02H63JZ, 02HK3JZ, 02HK4JZ |
| Renal replacement therapy | 5A1D70Z, 5A1D80Z, 5A1D90Z |
| Mechanical circulatory support | 5A02110, 5A02210, 5A0211D, 5A0221D, 5A02116, 5A02216, 02HA0QZ, 02HA3RJ, 02HA3RS, 02HA3RZ, 5A1522F, 5A1522G, 5A1522H, 5A15223 |

**Supplemental Table 2. Comorbidities of included patients according to valvular surgery status and stratified by risk of frailty**

| **Characteristic** | **Total** | **Low risk** | | **p-value** | **Intermediate risk** | | **p-value** | **High risk** | | **p-value** |
| --- | --- | --- | --- | --- | --- | --- | --- | --- | --- | --- |
|  |  | **Valvular surgery** | |  | **Valvular surgery** | |  | **Valvular surgery** | |  |
|  |  | **No** | **Yes** |  | **No** | **Yes** |  | **No** | **Yes** |  |
| **Weighted hospitalizations** | 53,275 | 19,895 | 2,875 |  | 21,990 | 6,305 |  | 1,620 | 590 |  |
| **Congestive heart failure** | 17,775 (33%) | 4,050 (20%) | 1,185 (41%) | <0.001 | 8,045 (37%) | 3,515 (56%) | <0.001 | 710 (44%) | 270 (46%) | 0.717 |
| **Cardiac arrhythmias** | 18,025 (34%) | 4,315 (22%) | 1,275 (44%) | <0.001 | 7,695 (35%) | 3,590 (57%) | <0.001 | 805 (50%) | 345 (58%) | 0.103 |
| **Valvular disease** | 29,360 (55%) | 10,395 (52%) | 2,130 (74%) | <0.001 | 11,210 (51%) | 4,470 (71%) | <0.001 | 745 (46%) | 410 (69%) | <0.001 |
| **Pulmonary circulation disorders** | 12,955 (24%) | 4,700 (24%) | 645 (22%) | 0.529 | 5,440 (25%) | 1,725 (27%) | 0.059 | 305 (19%) | 140 (24%) | 0.256 |
| **Peripheral vascular disorders** | 4,290 (8%) | 1,120 (6%) | 280 (10%) | <0.001 | 2,185 (10%) | 470 (7%) | 0.008 | 180 (11%) | 55 (9%) | 0.590 |
| **Hypertension** | 26,800 (50%) | 7,485 (38%) | 1,375 (48%) | <0.001 | 12,955 (59%) | 3,490 (55%) | 0.024 | 1,120 (69%) | 375 (64%) | 0.268 |
| **Paralysis** | 1,465 (3%) | 40 (0%) | 15 (1%) | 0.143 | 605 (3%) | 225 (4%) | 0.130 | 330 (20%) | 250 (42%) | <0.001 |
| **Other neurological disorders** | 6,925 (13%) | 945 (5%) | 165 (6%) | 0.303 | 3,715 (17%) | 1,170 (19%) | 0.169 | 730 (45%) | 200 (34%) | 0.036 |
| **Chronic pulmonary disease** | 9,755 (18%) | 2,990 (15%) | 420 (15%) | 0.792 | 4,660 (21%) | 1,235 (20%) | 0.216 | 360 (22%) | 90 (15%) | 0.108 |
| **Diabetes** | 11,915 (22%) | 2,925 (15%) | 440 (15%) | 0.704 | 6,330 (29%) | 1,495 (24%) | <0.001 | 550 (34%) | 175 (30%) | 0.396 |
| **Hypothyroidism** | 4,420 (8%) | 1,240 (6%) | 175 (6%) | 0.892 | 2,405 (11%) | 360 (6%) | <0.001 | 200 (12%) | 40 (7%) | 0.097 |
| **Renal failure** | 11,320 (21%) | 1,635 (8%) | 240 (8%) | 0.916 | 6,915 (31%) | 1,595 (25%) | <0.001 | 735 (45%) | 200 (34%) | 0.031 |
| **Liver disease** | 8,080 (15%) | 2,835 (14%) | 385 (13%) | 0.581 | 3,445 (16%) | 1,095 (17%) | 0.147 | 215 (13%) | 105 (18%) | 0.232 |
| **Peptic ulcer disease** | 320 (1%) | 65 (0%) | 10 (0%) | 0.934 | 155 (1%) | 70 (1%) | 0.153 | 20 (1%) | 0 (0%) | 0.224 |
| **AIDS/HIV** | 400 (1%) | 195 (1%) | 0 (0%) | 0.017 | 170 (1%) | 30 (0%) | 0.267 | 5 (0%) | 0 (0%) | 0.546 |
| **Lymphoma** | 365 (1%) | 80 (0%) | 15 (1%) | 0.677 | 205 (1%) | 35 (1%) | 0.198 | 30 (2%) | 0 (0%) | 0.135 |
| **Metastatic cancer** | 585 (1%) | 200 (1%) | 20 (1%) | 0.478 | 320 (1%) | 20 (0%) | 0.001 | 20 (1%) | 5 (1%) | 0.734 |
| **Solid tumor without metastasis** | 1,200 (2%) | 365 (2%) | 40 (1%) | 0.452 | 645 (3%) | 100 (2%) | 0.008 | 50 (3%) | 0 (0%) | 0.052 |
| **Rheumatoid arthritis/Collagen vascular** | 1,995 (4%) | 595 (3%) | 95 (3%) | 0.682 | 1,030 (5%) | 205 (3%) | 0.028 | 60 (4%) | 10 (2%) | 0.287 |
| **Coagulopathy** | 9,480 (18%) | 1,645 (8%) | 805 (28%) | <0.001 | 4,025 (18%) | 2,365 (38%) | <0.001 | 425 (26%) | 215 (36%) | 0.037 |
| **Weight loss** | 8,890 (17%) | 1,870 (9%) | 465 (16%) | <0.001 | 4,110 (19%) | 1,780 (28%) | <0.001 | 475 (29%) | 190 (32%) | 0.559 |
| **Fluid and electrolyte disorders** | 21,805 (41%) | 2,680 (13%) | 485 (17%) | 0.028 | 12,730 (58%) | 4,155 (66%) | <0.001 | 1,255 (77%) | 500 (85%) | 0.095 |
| **Blood loss anemia** | 610 (1%) | 130 (1%) | 35 (1%) | 0.136 | 295 (1%) | 120 (2%) | 0.144 | 20 (1%) | 10 (2%) | 0.712 |
| **Deficiency anemia** | 5,270 (10%) | 1,675 (8%) | 275 (10%) | 0.359 | 2,390 (11%) | 665 (11%) | 0.746 | 190 (12%) | 75 (13%) | 0.778 |
| **Alcohol abuse** | 3,520 (7%) | 1,030 (5%) | 165 (6%) | 0.572 | 1,635 (7%) | 550 (9%) | 0.131 | 105 (6%) | 35 (6%) | 0.834 |
| **Drug abuse** | 19,150 (36%) | 9,265 (47%) | 775 (27%) | <0.001 | 6,915 (31%) | 1,815 (29%) | 0.072 | 245 (15%) | 135 (23%) | 0.057 |
| **Psychoses** | 860 (2%) | 375 (2%) | 30 (1%) | 0.154 | 320 (1%) | 110 (2%) | 0.459 | 25 (2%) | 0 (0%) | 0.173 |
| **Depression** | 8,190 (15%) | 2,645 (13%) | 335 (12%) | 0.275 | 3,835 (17%) | 950 (15%) | 0.048 | 315 (19%) | 110 (19%) | 0.850 |

**
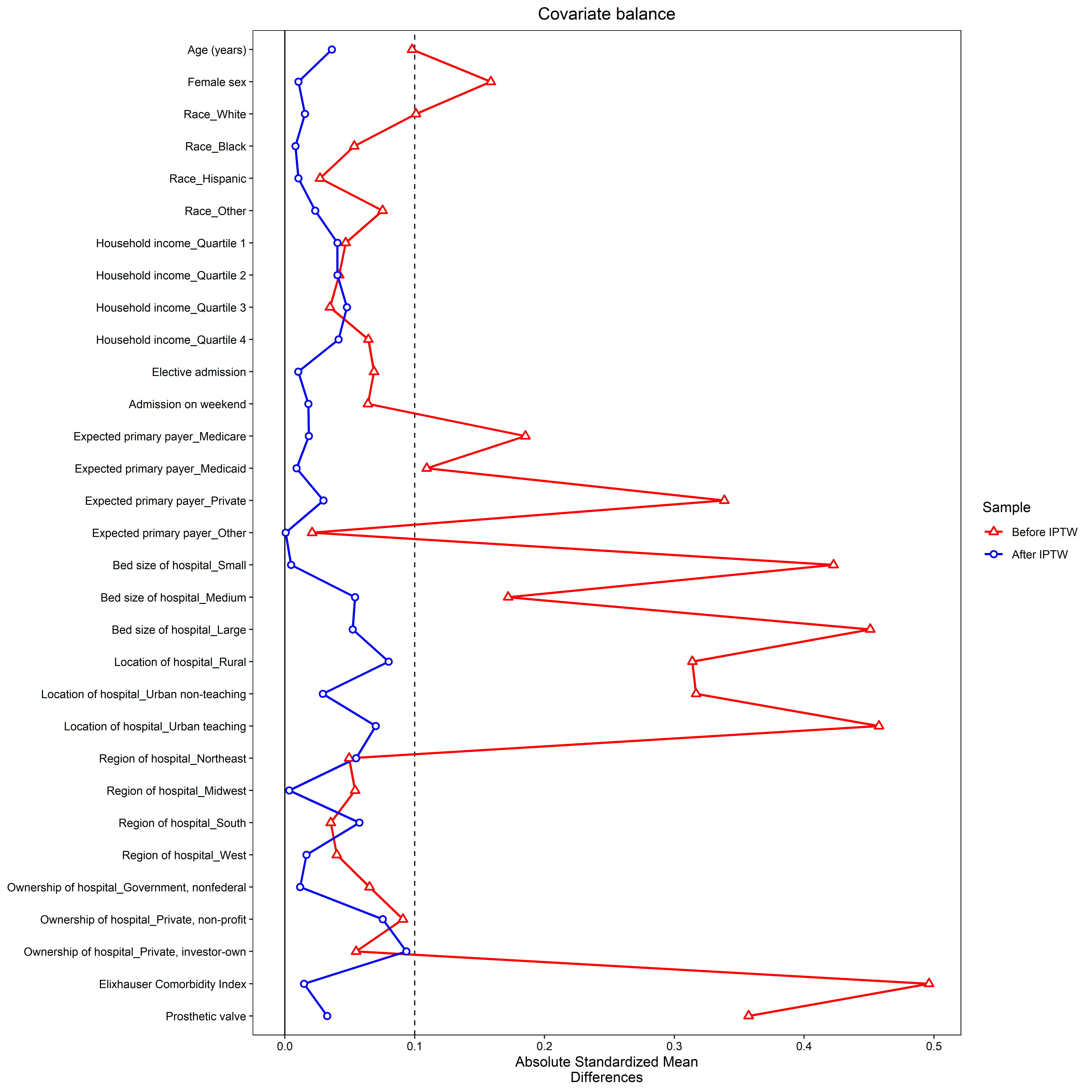
**

**Supplemental Figure 1. Balance in baseline covariates before (red line) and after (blue line) the inverse probability of treatment weighting.**
